# Differentiating Therapeutic Responses That Reduce Restrictive Practice Use and Situational Aggression in an Acute Mental Health Unit

**DOI:** 10.1111/jocn.17727

**Published:** 2025-03-14

**Authors:** Esario IV Daguman, Alison Taylor, Matthew Flowers, Richard Lakeman, Marie Hutchinson

**Affiliations:** ^1^ Southern Cross University Coffs Harbour New South Wales Australia; ^2^ Integrated Mental Health, Alcohol and Other Drugs Coffs Harbour Base Hospital Coffs Harbour New South Wales Australia

**Keywords:** cluster analysis, crisis intervention, de‐escalation, mental health services, physical restraints, psychiatric nursing

## Abstract

**Aim:**

An analysis of mental health nursing de‐escalation logs for 249 days from a regional adult inpatient unit in New South Wales, Australia, was completed to identify groups of cooccurring nursing therapeutic responses to aggression and examine their associations with reductions in restrictive practices and situational aggression.

**Design:**

A single‐centre retrospective study was undertaken.

**Method:**

Hierarchical clustering of nursing interventions established groups of cooccurring nursing responses. Poisson mixed‐effect models were then used to determine the associations of the intervention clusters with restrictive practices.

**Results:**

Two intervention clusters emerged: Cluster 1 involved verbal de‐escalation with active listening and rapport building, whereas Cluster 2 included additional limit setting and problem‐solving, distraction, sensory modulation, environmental change and individual staff time. Cluster 1 was linked with a reduction in seclusion use by 83% [IRR = 0.17, 95% CI (0.07, 0.41), *p* < 0.001], physical restraint by 79% [IRR = 0.21, 95% CI (0.11, 0.40), *p* < 0.001] and average judged situational aggression by 1.56 [95% CI (0.86, 2.25), *p* < 0.001]. Cluster 2 was related to statistically insignificant increases in the three studied outcomes.

**Conclusions:**

The intervention clusters prove the value of supplementary tools in surfacing nurses' therapeutic potential. The differences in restrictive practice use between intervention clusters signal the structure and progression of forming therapeutic relationships in aid of de‐escalation and the possibility of assessing de‐escalation components robustly.

**Relevance to Clinical Practice:**

Acknowledging and supporting nurses' therapeutic work support the development of recovery‐oriented care and a positive professional identity for nurses.

**Reporting Method:**

This study followed the applicable STROBE guidelines.

**Patient or Public Involvement:**

Due to the study's retrospective nature, there was no service user or public involvement.

**Summary**

- Daily mental health nursing de‐escalation logs provided real‐world insights into nurses' therapeutic responses to aggression.
- Clustering analysis identified distinct patterns in nurses’ responses to aggression.
- Stand‐alone verbal de‐escalation was linked to reduced seclusion and restraint in an acute mental health service.
- Applying limit setting and individualised staff time to a set of nursing interventions may yield an insignificant increase in restrictive practices.

## 1 Introduction

Within the remit of acute mental health nursing practice, access to dependable and enduring relationships is vital for supporting recovery. These relationships thrive on skilfully applying relational competence, a concept described as being good at understanding and managing oneself, genuinely caring about understanding the service user,^1^ interacting with them fairly and respectfully and ensuring they are valued and important (Beyene et al. 2024). Capturing the dynamics of these competencies alongside the quality of nurse–service user relationships ensures that support is individualised and service user involvement and choice are maximised (Stanhope and Matthews 2019). Yet, this central task is not easy. Records in mental health units of nurses' interventions and their quality within a relationship have not been sufficient, despite available nursing documentation standards (Pérez‐Toribio et al. 2024).

Thoughtful documentation scaffolds the emergence of acute mental health nursing practice, which is often unseen and may benefit from role clarification (Terry 2020). They support access to essential earlier interactions with, and clinical information of, service users (Martin et al. 2023). Good documentation helps identify behavioural change mechanisms behind interventions to reduce restrictive practice use in adult mental health inpatient settings (Baker et al. 2021). Restrictive practices, such as seclusion and restraint, are reportable coercive incidents that impinge on individual autonomy and are considered ‘failures of care’ (Savage et al. 2024, 1). They are monitored differently worldwide.

Clinical nursing notes are one way of articulating interventions within the therapeutic relationship. However, they do not always reflect what they were intended to be. Sometimes, they are deficit‐oriented and considered mere summaries of the service users' activities in a shift (Myklebust and Bjørkly 2019). While objective, such documentation quality may reinforce a limited view of mental health nurses as observers and custodians, which in turn does not promote service users' autonomy over their safety and recovery. When nurses are not supported to see the impact of thoughtful documentation on service user care, it may demotivate them to document thoughtfully and hinder the development of a positive professional identity (Rossi et al. 2023) outside of debatably limiting practice scopes.

Nurses' ability to influence the pathways to coercive adult acute inpatient care is limited. Of 124 articles screened in an overview of reviews, four reviews reported that the impact of the interventions nurses can deploy to reduce aggression, conflict and restrictive practices is mixed, specifically for staff training and sensory rooms and equipment (Daguman et al. 2024). Research evaluating intervention effectiveness has typically focused on multicomponent programmes that include relational competencies, such as the Safewards' ‘Talk Down’ (Bowers et al. 2015) with delimiting, clarifying and resolving, and the six core strategies (Huckshorn 2004, 2005) with trauma‐informed debriefing. These programmes have been linked to reductions in aggression, interpersonal conflict and restrictive practices (Allen et al. 2018; Finch et al. 2021; Gaynes et al. 2017). However, a major limitation of this body of work is identifying which programme elements contributed the most to the overall effect. Importantly, any effect of nurses' therapeutic responses has not been evaluated in isolation.

A similar gap exists in interventions on de‐escalation, which encompass a range of relational competencies such as emotional self‐regulation, distress validation and collaborative problem‐solving, all aimed at bringing escalations to a safe space (Price, Armitage, et al. 2024). Of 2774 papers screened in a review, 46 studies of primarily poor quality focused on the effectiveness of de‐escalation training, rather than relational competencies (Price, Papastavrou Brooks, et al. 2024). The outcomes regarding conflict and the use of restrictive practices were inconsistent. Cognitive, affective and skills‐based outcomes were evaluated, with limited consideration of situational aggression—the immediacy of a conflict that escalates through stages of increasing emotional arousal and aggressive behaviours (Dickens, O'Shea, et al. 2020). This limitation conflicts with the idea that most violent events within and beyond mental health settings arise from escalating situations (Lavelle et al. 2024).

Broadening evaluations of de‐escalation interventions and teasing out their active components help optimise their impact and better personalise mental health support, consistent with the goals of dismantling studies on cognitive–behavioural therapies and the widely held belief that service user characteristics influence therapy outcomes (Furukawa et al. 2021). There is evidence that impersonalised de‐escalation approaches are perceived as heightening a person's anger (Spencer et al. 2021). More importantly, evaluating relational competencies means appreciating the work of mental health nurses, who value these competencies when supporting people in crises (Wilson et al. 2024). In a time of growing mental health needs, their potential must not remain underrecognised and underutilised (Hurley et al. 2022). Mental health nurses are not only service user advocates and physical health therapists but also psychotherapists, psychopharmacological therapists, aggression management therapists and relationship‐focused therapists (Hurley and Lakeman 2021).

At an acute mental health unit in New South Wales (NSW), Australia, a gap in documenting and evaluating valuable interventions within therapeutic relationships led a clinician (MF) to design a supplementary paper‐based daily mental health nursing de‐escalation log in 2019. Over 30 months, the log captured nursing de‐escalation practices, providing a reference for clinical notes and informing restrictive practices review meetings and nurse debriefings. Another clinician (AT) later refined the log to support educational programme development within the unit. These efforts aligned with broader reforms in NSW (NSW Health 2017), which enhanced accountability for using restrictive practices.

Maximising the potential of mental health nursing de‐escalation log data through cluster analysis can help derive the optimal combination of interventions in reducing restrictive practices and situational aggression. Real‐world data, such as the log data, can be repurposed to supplement traditional knowledge based on the comparative effectiveness of interventions (Berger et al. 2017). Cluster analysis is an accessible option for discerning active components of therapeutic interventions usually undertaken through costly experimental approaches (Leijten et al. 2021). The clustering of real‐world data on mental health nursing interventions to contain conflicts has been exemplified in the past (Bowers et al. 2006). However, there was no clustering of relational competencies and noncoercive interventions. There was also no subsequent evaluation of their differential associations with restrictive practice use and situational aggression.

This study was intended to address the gaps in the broader evaluation of, and identification of active components in, groups of cooccurring strength‐based mental health nursing responses to aggression. The current investigation was an exploratory reanalysis of existing de‐escalation logs envisioned to provide a new empirical understanding of and generate hypotheses for, the relationship between the emerging clusters and restrictive practice use, physical injuries and situational aggression. Secondly, this reanalysis was aimed at deriving the minimum sample size requirements for evaluating the between‐clusters differential associations.

## 2 Methods

### 2.1 Design

A single‐centre, retrospective study was undertaken to repurpose daily de‐escalation logs completed by nurses over 30 months (January 2019 to May 2021) within a declared acute mental health unit in NSW, Australia. Unlike earlier studies that relied on official incident reports completed after individual events (Bowers et al. 2015), this study used daily aggregates of mental health nursing de‐escalation logs as the unit of analysis. An aggregated approach avoids the illusion of completeness and allows a more accurate assessment of potential reporting biases in real‐world data. This study is the second in a series of retrospective studies that examine aggression and restrictive practices in a unit. The prepandemic estimations and third‐party data processes described in this Methods section are similar to those of the other two studies. However, they have different purposes and quantitative approaches. This study followed the applicable Strengthening the Reporting of Observational Studies in Epidemiology guidelines (STROBE; Vandenbroucke et al. 2007) for reporting observational studies.

### 2.2 Data Source and Sampling

The logs were sourced from a 30‐bed adult acute mental health facility in a public teaching hospital, including a few high‐observation beds. It operates under Australia's universal healthcare framework, which aligns with the goals of Universal Health Coverage (WHO 2023), ensuring access to free, government‐funded mental health support. The facility emphasises least restrictive care, with inpatient admission considered a last resort. The unit operates 24 h a day and is staffed by a multidisciplinary team, including nurses, a clinical nurse educator and a nurse educator, providing varying mental health treatment and observational support.

**FIGURE 1:**
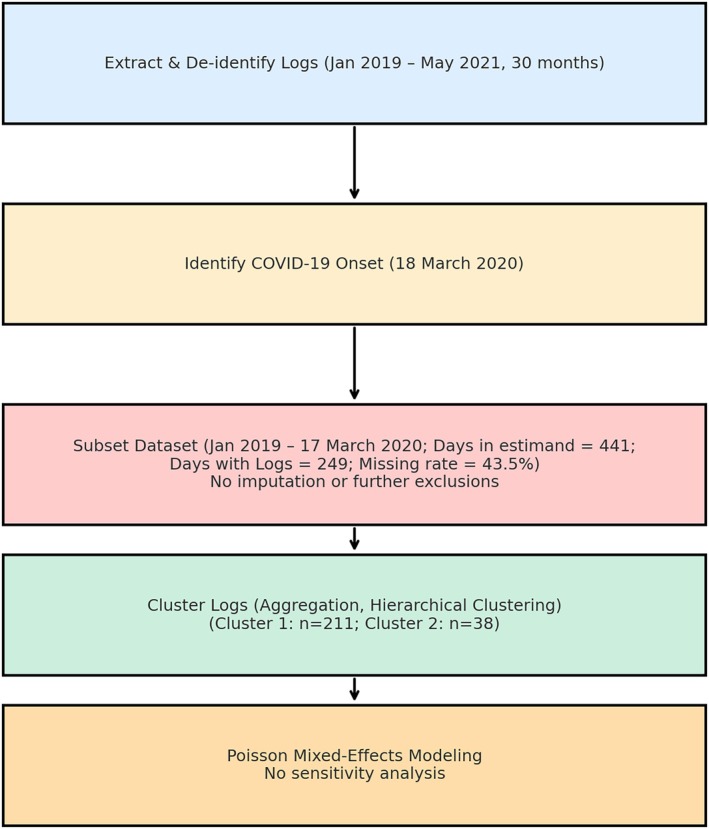
Process flow of study procedures. [Colour figure can be viewed at wileyonlinelibrary.com]

Only a subset of logs from the 30 months was analysed. Pragmatic adjustments were made to distinguish the associations of nursing intervention clusters on restrictive practice use from those related to the COVID‐19 pandemic. These associations were assessed through a prepandemic estimand (Cro et al. 2020). Clinical input identified the 18 March 2020 as the pandemic's onset within the studied inpatient unit, which coincided with the state's easing of work restrictions for student nurses mobilised for the pandemic response and placed a new demand on the clinical nurse educator and nurse educator to focus on their training. All available completed logs from the onset of the pandemic until May 2021 were then set as missing and unnecessary for this study. Of the 441 days within the prepandemic estimand, 249 days have completed log data (or 917 logs). The resulting missingness rate (43.54%) indicated the application of another established guideline that recommends against multiple imputations on datasets with more than 40% missingness (Jakobsen et al. 2017). This approach helps prevent potential downward bias from imputing data without a true reference value, as nonevent days were treated as missing data in the study and restrictive practice outcomes can have a discrete and skewed distribution. No exclusion criteria were applied at the log‐level analysis. Figure 1 summarises the process of deriving the total number of logs analysed.

Model‐based retrospective power analyses were performed through Monte Carlo simulations in the simr package (Green et al. 2023) and RStudio (Posit Team 2023) to inform future evaluations. Synthetic data were generated to replicate the maximum observed values in the logs and the corresponding mixed‐effect model specifications described in the Data Analysis section. The simulations indicated that a 6‐month data collection period for two comparison groups is necessary to detect a 12% reduction in seclusion use with 94% power, aligning with the effect size in an NSW Safewards study (Dickens, Tabvuma, et al. 2020). Eight months is required to detect similar effect sizes for physical restraint (95% power) and eight and a half months to achieve a 1.55 beta increase in situational aggression change pre‐ and post–de‐escalation (86% power).

### 2.3 Measure

The mental health nursing de‐escalation log is structured into five main sections: (i) incident details, (ii) aggression type, (iii) interventions or responses, (iv) judged situational aggression levels and (v) outcomes, encompassing 31 variables. The first section captures contextual information, including the date, location of the incident and the number of nurses involved in de‐escalation. The second section classifies aggression incidents regarding their direction (e.g., service user to nurse aggression). The third section documents various responses, including verbal de‐escalation, distraction, sensory modulation, reality basing or testing, change of environment, individualised staff time, phone calls, culturally sensitive care and hospital ground leave. Descriptions of these interventions are provided in the Supporting Information. The verbal de‐escalation intervention included two coded relational skills: ‘active listening and rapport‐building’ and ‘limit‐setting and problem‐solving’.

The fourth section features a 6‐point scale to evaluate situational aggression before and after de‐escalation efforts. The scale ranges from a score of 1, representing a state of calm, to 2, indicating agitation characterised by pacing, shaking, restlessness or rocking. A score of 3 denotes verbal aggression, manifesting through abusive language, insults or threats. A score of 4 reflects physical aggression directed towards property. In contrast, 5 represents physical aggression towards others or oneself, including actively combative behaviour. The highest score, 6, signifies physical aggression involving weapons. Finally, the fifth section records any bodily injuries and restrictive practice use, including the implementation of seclusion and physical restraint.

Nurses received training from educators on completing the logs for every incident of aggression and mental health crisis they have responded to. There were no specific inclusion criteria for whom the logs were to be completed. They were cross‐referenced with official unit registers and administrative data to reduce potential recall bias. Total counts of a range of outcomes can be derived by summing up the observed events. To ensure concurrent validity, the total daily numbers of the outcomes section variables were compared with those in the unit's administrative data that underwent checks for state‐wide reporting. A similar methodology to Bowers (2008) was used for validating only the days on which both datasets had comparable information. There was a strong concurrence between the datasets for the seclusion [*r* = 0.76, 95% CI (0.62, 0.88), *p* < 0.001] and physical restraint [*r* = 0.82, 95% CI (0.73, 0.90), *p* < 0.001] outcome data.

### 2.4 Procedures

The data custodian authorised a nominated staff member to source and de‐identify the data. This process ensured that all potentially identifiable information was removed from the data before analysis to safeguard participant privacy. It was conducted in adherence to data privacy regulations. The first author, blinded to the identity of the nurses who completed the logs, aggregated outcome data and conducted the analysis to minimise bias in measuring the outcomes. Figure 1 depicts this study's procedures, from data extraction to analysis. Under the National Statement on Ethical Conduct in Human Research (NHMRC 2023), the data custodian institution has an established process for reviewing low‐ and negligible‐risk research and determining whether it requires Human Research Ethics Committee (HREC) approval. The study was assessed and exempted from ethics review (Reference Number: QA2024_05), as it involves using existing data collections or records that contain only nonidentifiable data about individuals (NSW IPC 2019) and is a negligible‐risk research (NSW OHMR 2023). A relevant university provided full HREC ethical approval for this study (Approval Number: 2024/145).

### 2.5 Data Analyses

The data underwent a two‐phase statistical treatment. Inductive hierarchical clustering analysis (HCA) was first performed. HCA is an unsupervised learning method that groups data based on proximity (Aldenderfer and Blashfield 1984). In this study, HCA was used to measure the proximity between rows of daily intervention data for identifying groups of cooccurring patterns in nurses' responses to service user aggression. The closest data pairs were merged iteratively, and proximity between data points was recalculated as clusters formed. Mixed effects were then modelled to explore how these patterns influenced outcomes.

In the HCA, the intervention or response data were transformed using log1p to handle zero‐inflated counts and then scaled for normalisation. Data were subsequently clustered using complete linkage, which merges clusters based on the maximum distance, and Canberra distance, suited for nonnegative values like counts (R Core Team 2024). Dendrograms were graphed and cut by the optimal number of clusters determined using a silhouette plot (Rousseeuw 1987), demonstrating the cohesion within and separation from other clusters. The daily aggregate of interventions was assigned to a cluster in the dendrogram. A cophenetic correlation coefficient (CCC) was used to assess the fit between the original dissimilarity and simplified clustering matrices, where 0.7 or higher indicates the adequacy of the clustering method for summarising the dataset (Rohlf 1970).

To analyse aggregate‐level differences in seclusion, physical restraint and physical injuries among emerging clusters, Poisson mixed‐effects models were fit using maximum likelihood and bound optimisation by quadratic approximation optimiser in the lme4 package (Bates et al. 2022). These models allowed for handling count data by modelling fixed predictors and hierarchical random effects, thus accommodating overdispersion and individual‐level differences (Cameron and Trivedi 2013). Overdispersion and excess zeroes were checked to confirm the appropriateness of Poisson over negative binomial mixed models (Hilbe et al. 2013). The models included two random effects—the pre–de‐escalation situational aggression categories nested within months for both seclusion and physical restraint and the post–de‐escalation situational aggression categories for seclusion only. This model specification was informed by imminent aggression (Dickens, O'Shea, et al. 2020) and the established lack of associations between service user‐related influences and conflict and containment (Papadopoulos et al. 2012). The coefficients of determination, R^2^, were calculated using the delta approach (Nakagawa et al. 2017).

On the other hand, linear mixed‐effects modelling was undertaken to determine the change in situational aggression levels before and after de‐escalation between clusters, and the years nested within months were considered the random effect. The figures in this study were created using Matplotlib (Hunter 2007). Sensitivity analyses were also avoided to prevent disrupting the clustering that underpins the fixed effect in the mixed modelling.

## 3 Results

**FIGURE 2:**
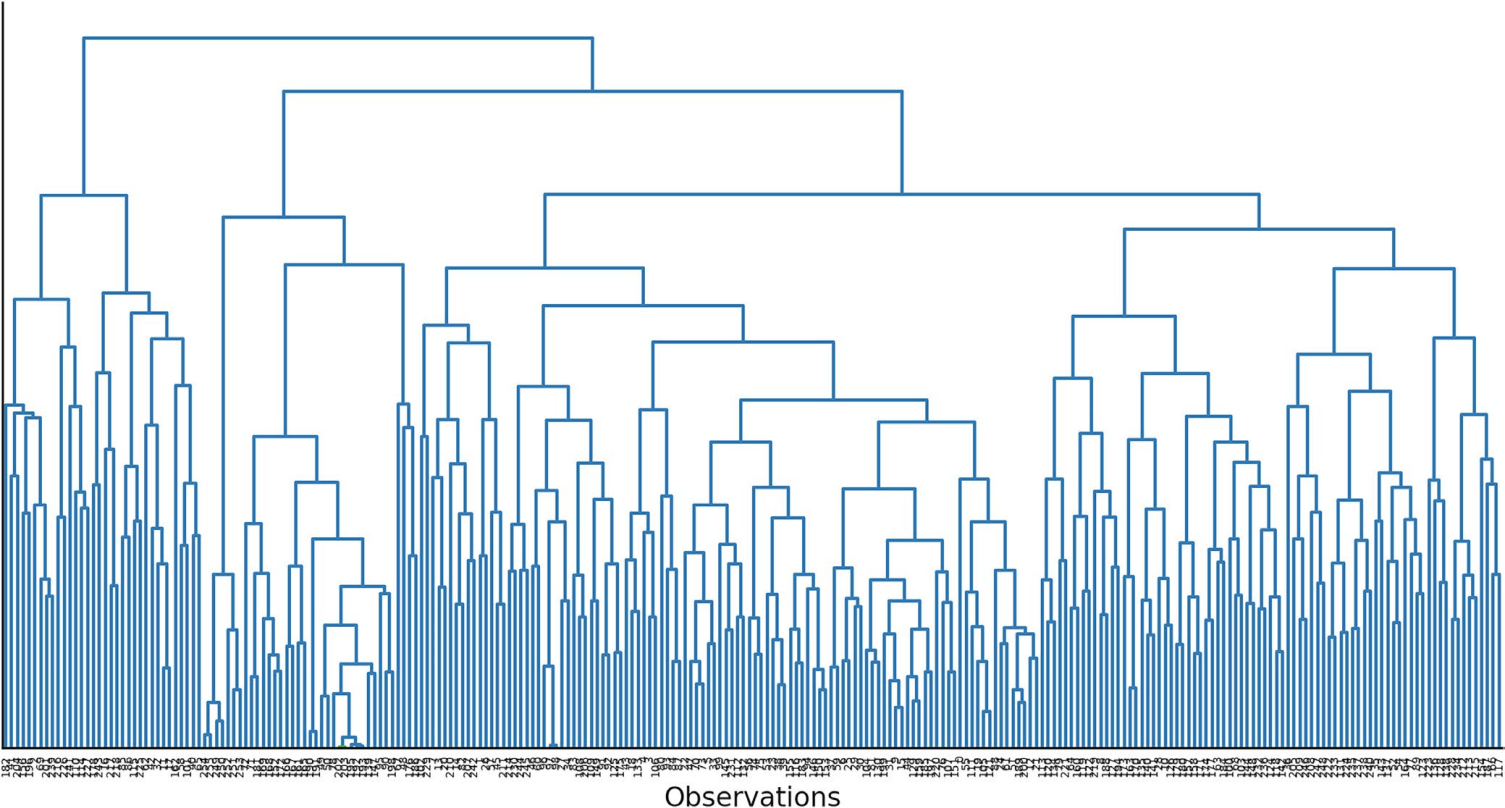
Dendrogram of daily aggregates of nursing interventions. [Colour figure can be viewed at wileyonlinelibrary.com]

**FIGURE 3:**
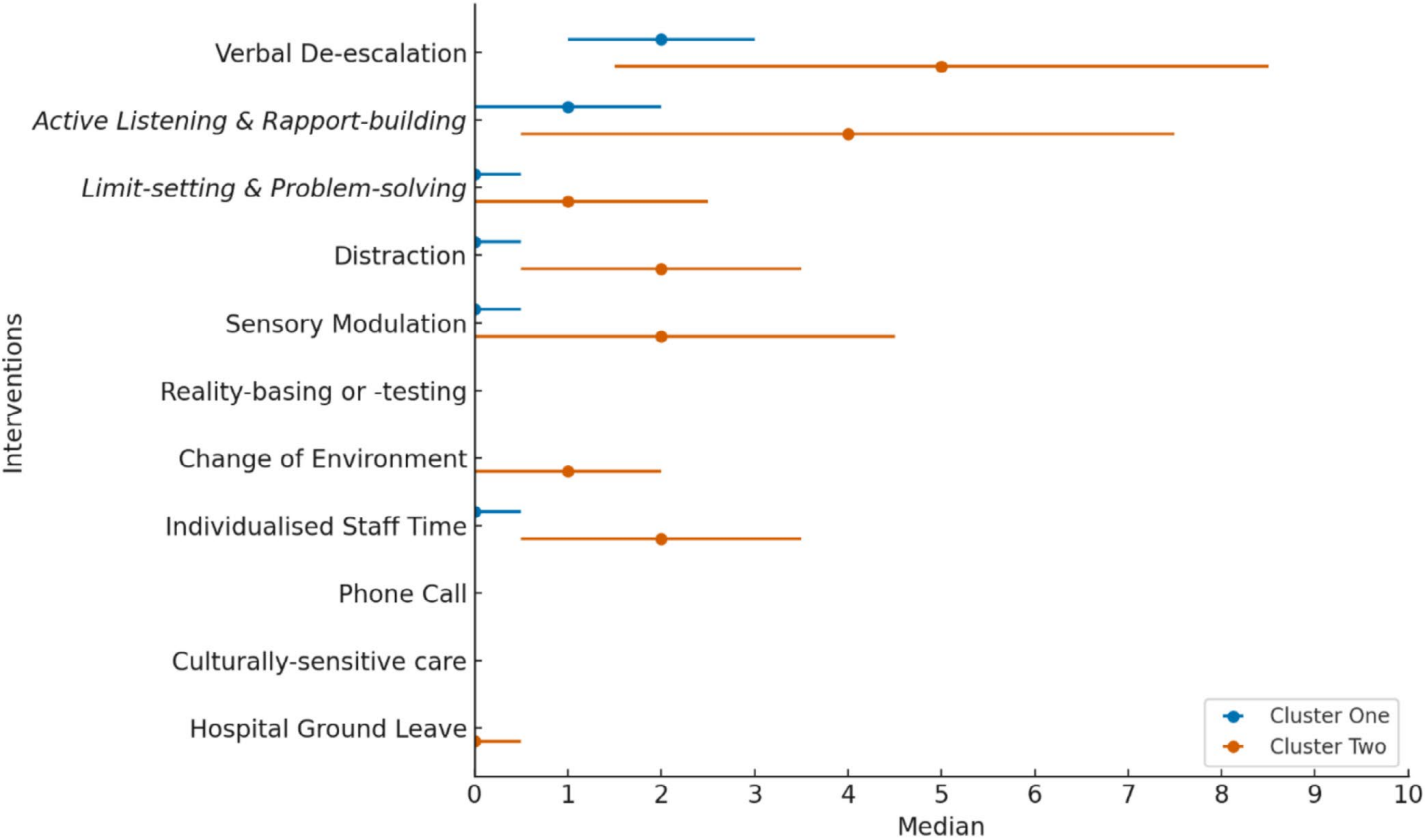
Medians and interquartile ranges of daily nursing interventions between clusters. [Colour figure can be viewed at wileyonlinelibrary.com]

The silhouette plot demonstrated the highest average silhouette coefficient for two clusters, suggesting that the optimal data partitioning is attained with two distinct clusters (see Supporting Information). The dendrogram of daily intervention aggregates was then partitioned into two clusters: the broader cluster branching out of the top right side of the graph represents Cluster 1 (85%; *n* = 211), while the narrower left branch represents Cluster 2 (15%; *n* = 38; see Figure 2). Figure 3 describes the clusters, showing daily nursing interventions' medians and interquartile ranges. Cluster 1 included stand‐alone verbal de‐escalation, while Cluster 2 formed a distinct cluster composed of additional interventions such as distraction, sensory modulation, change of environment and individualised staff time. Clustering was extended to datasets on coded relational skills. This analysis indicated that active listening and rapport building were applied in Cluster 1. In Cluster 2, the application included active listening, rapport building, limit setting and problem‐solving. The CCC was 0.78, indicating a solid fit between the dendrogram and the original dissimilarity matrix.

Poisson mixed‐effects models were fitted to investigate the relationship between clusters of nursing interventions and restrictive practice use and physical injuries. The restrictive practice use models did not show overdispersion and excess zeroes. However, singular fits were observed for the models on nurse and service user physical injuries, which had low counts throughout the study period (11 incidents for service users and eight incidents for nurses). Consequently, the physical injury outcomes were excluded from the mixed effects modelling, while the models for seclusion and physical restraint had low explanatory power (conditional R^2^ = 0.18–0.20).

**FIGURE 4:**
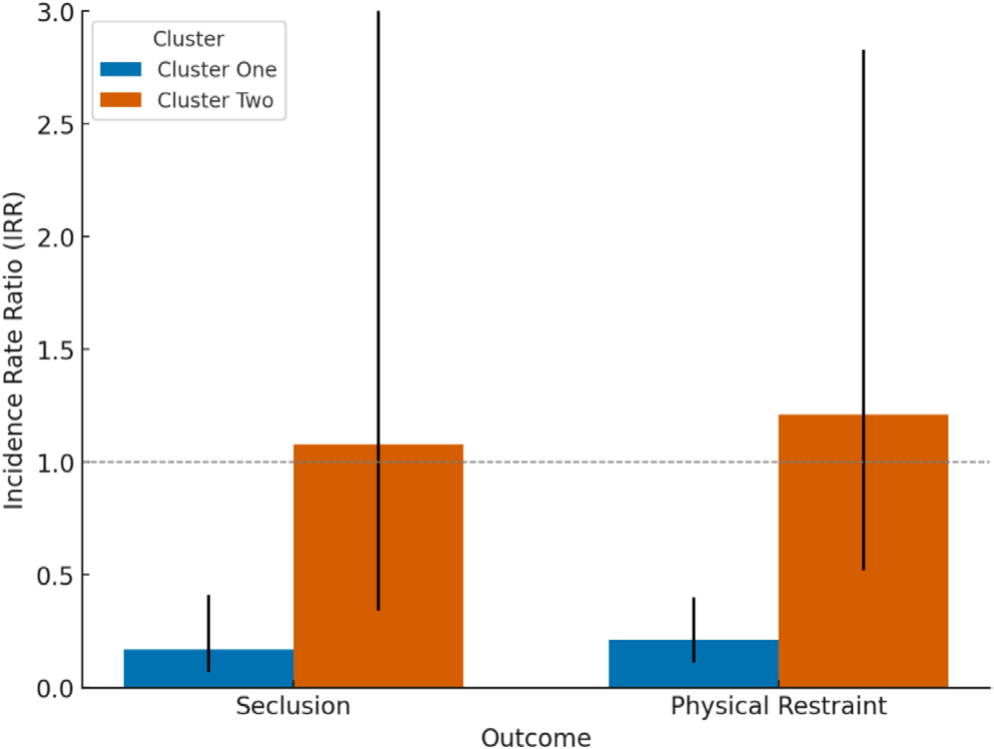
Between‐group differences in incident rate ratios of restrictive practice use at 14 and a half months, with 95% confidence intervals. [Colour figure can be viewed at wileyonlinelibrary.com]

After accounting for the judged situational aggression categories before and after de‐escalation, as well as the month of the daily incident aggregates, Cluster 1 had a statistically significantly lower incidence of seclusion events (incident rate ratio (IRR) = 0.17, 95% CI [0.07, 0.41], *p* < 0.001, adjusted intraclass correlation coefficient (ICC) = 0.34, conditional R^2^ = 0.18 and marginal R^2^ = 0.0002) compared to Cluster 2 (IRR = 1.08, 95% CI [0.34, 3.41], *p* = 0.90; see Figure 4). Similarly, Cluster 1 demonstrated a significantly lower rate of physical restraint use (IRR = 0.21, 95% CI [0.11, 0.40], *p* < 0.001, adjusted ICC = 0.35, conditional R^2^ = 0.20 and marginal R^2^ = 0.001) than Cluster 2 (IRR = 1.21, 95% CI [0.52, 2.83], *p* = 0.65). The physical restraint model had the pre–de‐escalation situational aggression levels and the month of the daily aggregates set as random effects.

**FIGURE 5:**
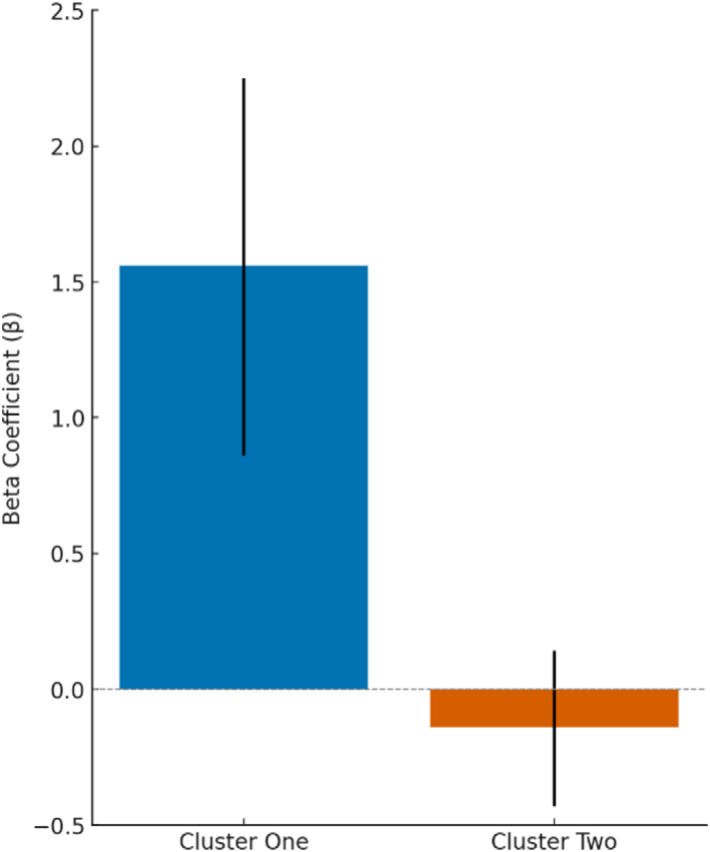
Between‐group differences in average judged situational aggression before and after de‐escalation, with 95% confidence intervals. [Colour figure can be viewed at wileyonlinelibrary.com]

The results of the linear mixed model revealed a significant reduction in average judged situational aggression in Cluster 1 [*β* = 1.56, 95% CI (0.86, 2.25), *p* < 0.001; see Figure 5] while accounting for the month and year of the daily incidents aggregate. In contrast, for Cluster 2, there was a slight, statistically nonsignificant increase [*β* = − 0.14, 95% CI (−0.43, 0.14), *p* = 0.32]. The direction of these coefficients reflects the subtraction of post–de‐escalation aggression levels from pre–de‐escalation levels. The model's explanatory power was almost moderate (conditional R^2^ = 0.28).

## 4 Discussion

Verbal de‐escalation was the often‐used intervention, with occasional implementation of distraction, sensory modulation, a change in environment and individualised staff time. This finding contrasts with a retrospective study on nurses' notes that primarily emerged as notetakers focus on physiological interventions, rather than relational ones (Pérez‐Toribio et al. 2024). The log used in the current study makes the central difference; it was socialised within the unit to emphasise relational interventions and was supplementary. This difference suggests that such focus on documentation and training for using a measure can influence what nurses employ as interventions, and an obvious space for tools that can hold a picture of the psychotherapeutic nurse exists. This finding seems to be associated with minimal social desirability bias, as shown by the variability of intervention use across clusters.

The importance of relational interventions in managing aggression finds support in the emergence of the clusters, showing the alignment of the nurses' knowledge and behaviours with existing policy and research for preventing violence in mental health settings (NSW Health 2015; Price, Papastavrou Brooks, et al. 2024). The clusters solidify the progression in forming therapeutic relationships, similar to Peplau's proposal (Forchuk 2021). De‐escalation unfolds on a timeline, starting with holding space for service users to share and feel heard. Nurses then shape work towards service users' goals by helping them process their issues, adjust to sensory input from the environment or actively change their surroundings.

The findings demonstrated a statistically significant link between applying stand‐alone verbal de‐escalation and decreased seclusion, physical restraint and situational aggression. However, external validation of these studies is limited by the aforementioned focus of intervention evaluations on the impact of de‐escalation training (Leach et al. 2019). A recent study in Slovenia exemplifies this challenge. Despite the study being presented in its title as the ‘effectiveness of de‐escalation’ (Celofiga et al. 2022, 1), the reduction in the incidence and severity of aggression and physical restraint use observed were associated with the de‐escalation training, with a protocol that includes active listening, limit and rule setting and distraction as techniques. There was no determination on whether a technique in isolation or a specific combination drove these reductions. It follows that latitude for assessing the quality of evaluations for de‐escalation be given primacy.

The value placed on active listening and rapport‐building and the introduction of restrictive practice review meetings in the unit may explain the associated outcomes in Cluster 1. Active listening has been recognised across various care settings as a key means to understanding what a service user is expressing (Pratt et al. 2021). It differs from listening to respond, which overlooks the opportunity to hear the service user and show authentic concern for them. Building rapport places service users at ease. It signals acknowledgement of what they present and encourages them to share more. On the other hand, the restrictive practice review meetings facilitated by a nurse unit manager (PG) involved regular examination of log data at each meeting and reflections about systemic influences on incidents of aggression and restrictive practices. There is evidence for the effectiveness of postrestrictive practice debriefing and review in reducing seclusion and improving therapeutic relationships (Goulet et al. 2018).

The outcome reductions seen in Cluster 1 further show that positive findings are linked with using a subset of relational competencies. It is common for people in distress to seek emotionally resonant individuals to feel understood (Ekman and Ekman 2017). In contrast, others may not know their needs yet. Coexisting relational techniques in a structured de‐escalation can then be partially deployed without compromising their purpose. However, nonrelational interventions begin only after their relational counterparts. Discussion of the next steps is postponed until later in a de‐escalating conversation because taking action can sometimes feel impossible and suggesting it early on can seem coercive or dismissive. This finding may also explain why some interventions with fixed or prescribed language within the Safewards model feel patronising and condescending (Ward‐Stockham et al. 2022). Although useful for beginners, such language may seem robotic and insincere.

On the other hand, Cluster 2 was related to insignificant increases in seclusion, physical restraint and situational aggression. These results conflict with reduced restrictive practice use in quantitative evaluations of multicomponent interventions with collaborative problem‐solving (Perers et al. 2021) and sensory approaches (Oostermeijer et al. 2021), and accounts in qualitative studies where service users and healthcare professionals consider distraction techniques as an alternative to physical restraints (Kim and Nam 2024). A putative mechanism for the Cluster 2 outcomes could be the use of limit setting and individualised staff time. Both limit setting, which can be viewed as a milder form of coercion (Vatne and Fagermoen 2007), and one‐to‐one nursing have been individually linked with an increased likelihood of aggression in an archival study within an Australian forensic mental health service (Maguire et al. 2018). However, the statistical significance varied between the genders of nurses employing them. In the current study, a service user's distress may have been elevated by limit setting and individualised staff time, ushering them to display a response thought to be such that restrictive practices should be applied. The Cluster 2 outcomes are not representative of the responses in the unit, as it has a small sample size.

The Cluster 2 interventions further depicted the complexity of service users' needs and cautions of the reasoning bias that can arise from overemphasising nurses' relational skills. Since escalations are as individual as de‐escalations, nurses self‐organise by involving other techniques for service users to make sense of their crisis (Tucker et al. 2020), including providing outlets for expressing their experiences. These additional interventions can provide a buffer from overly valorising relational skills that deflect a service's responsibility of solving systemic issues (Virkki 2008). Fundamental issues of coercion and othering can be occluded when nurses' relational skills are framed as resilience against aggression. In contrast, other causes of systemic issues exist, and a multiple‐model view of solutions is needed. Learning then from Abraham Maslow's (1966) words may be relevant: ‘I suppose it is tempting, if the only tool you have is a hammer, to treat everything as if it were a nail’ (p. 15).

Taken together, the study findings provide quantitative evidence of verbal de‐escalation's effectiveness, although the key message is not numerical. The findings show that minimising the impact of aggression within a unit involves maximising the relational competencies of nurses. These findings challenge traditional views of nurses being treated as tools, who carry out delegated or instrumental work for others (Lakeman et al. 2023). Nurses cultivate relationships that can refashion their professional identity beyond subservience. They can conscientiously object to restrictive practices and promote human rights; nonetheless, they need organisational support (Gadsby and McKeown 2021).

If nurses are to be supported to reach their full potential, a deliberate effort must then be made to make their skills active. Unfortunately, notwithstanding calls for equal focus, nursing education and regulation often overemphasise physical health, neglecting unique mental health needs (Warrender et al. 2024). In Australia, there has been insufficient attention to specialist undergraduate training and the development of relational skills in the mental health nursing workforce (Hurley et al. 2024). Additionally, in a time of high demand for mental health support, credentialed mental health nurses in Australia are often excluded from major funding programmes that provide service users access to their psychotherapeutic services (Lakeman et al. 2020). Compare this status quo to how such a plea for support would be answered in a nonmental health nursing space; there could be an undue contrast.

Another barrier is the tension between the lack of thoughtful documentation of nursing interventions and increasing time spent on record keeping. Nurses' contributions in elaborating work towards service user goals have been less articulated and often seen as subpar (Myklebust and Bjørkly 2019). This underrecognition of nursing contributions can obscure nurses' valuing of, and confidence in, the profession (Terry 2020). On the other hand, the time spent on record taking may prevent mental health nurses from authentically relating to service users (McKeown 2024). This tension then begs to ask about nurses' psychological safety when attempting thoughtful documentation and the potential lowering of expectations that increase their sole focus on the physical health dimension of mental health nursing. Nevertheless, nurses often do remarkably rise to the challenge.

### 4.1 Recommendations for Further Research

Avenues for future research follow. Firstly, the study employed a prepandemic estimand to analyse data unaffected by indirect participant behaviour changes, such as increased nurse workload due to retraining. Interpreting the estimand's outcomes remains challenging because the extent of the prepandemic missingness still exceeds reasonable imputation limits. This missingness rate may be due to the log's supplementary nature and the busy environment of acute care units. Any additional minor tasks might have been a big ask from the nurses (Bowers et al. 2015). As such, the findings need to be considered hypothesis generating.

The present study showed low‐ to almost moderate‐conditional R^2^ values for the models on seclusion, physical restraint and situational aggression. Although a low value of at least 0.1 is adequate in social science research (Ozili 2022), the observed values may be due to this study's single‐ward setting that limited the variables available for model nesting and the complex nature of the outcome variables.

The format and purpose of the log used in the study have limited the range of analysed interventions, the determination of time spent deploying relational competencies, the identification of personal characteristics of the nurse participants and the covariates for statistical model specifications. Characteristics of service users involved in incidents of de‐escalation were also not recorded in the logs. However, many acute care reform evaluations often do not report diagnostic information (Daguman et al. 2024). Future studies may consider introducing open response fields in the logs for time stamps, other interventions and service user and nurse information to help determine what new or existing intervention cluster works best with a specific demographic. Furthermore, a considerable case exists for examining the contribution of high involuntary treatment context in Australian public acute mental health units—46% of hospitalisations in 2019–2020—which are highest among males, people aged 35–39 and people diagnosed with schizophrenia and schizoaffective disorders (AIHW 2024).

The de‐escalation log has been validated, focusing on the concurrent validity of the outcome section. These psychometric properties matched the assessment purpose for which the log was used, given the retrospective nature of this study and the criteria for recognising restrictive practice use are clear, less subjective and highly regulated. End users of measures need to first identify the measure's purpose and underlying concept before validating any properties (Daguman and Taylor 2024). With these priorities addressed, other properties need to be examined, including content validity ratio and interrater reliability.

Finally, using standardised measures—with a balance of strengths and areas for improvement—can make the impacts of nursing interventions more visible. Developing information on additional outcomes through these standardised measures can support the national priority of reporting on indicators that contribute to evaluating mental health service performance in Australia (NMHPSC 2013). It may then be helpful to consider replicating the findings against other restrictive practice outcomes, such as durations of seclusion and physical restraint.

### 4.2 Implications for Practice

The study findings amplify the message of basic competencies: being available in crisis through active listening and rapport building is a way for nurses to support the development of therapeutic relationships and reduce the use of restrictive practices within a unit. Nurses can role model skills for service users to take personal responsibility in managing distress through these engagements. Emotional responses in these engagements can sometimes be hard to manage, and a structured yet flexible approach can help nurses regain focus in bringing service users to a safe space. Nurses can further shape their everyday provision of acute care through reflective practices and recording tools that surface their therapeutic potential. Without actionable evidence of this potential, the natural tendency may be to focus on physiologically based nursing interventions.

## 5 Conclusions

The analysis of mental health nursing de‐escalation logs revealed two clusters of nursing responses to aggression in an acute care unit. Stand‐alone verbal de‐escalation was the often‐used intervention. It was significantly associated with reduced seclusion, physical restraint and situational aggression. When combined with other nursing interventions, verbal de‐escalation was linked with a nonsignificant increase in physical restraint events and seclusion use. Further research is needed to evaluate these interventions against other mental health service performance indicators, utilising a log that has undergone further psychometric validation and a larger dataset.

## Author Contributions

**Esario IV Daguman:** conceptualisation, methodology, software, formal analysis, investigation, resources, data curation, writing – original draft and visualisation. **Alison Taylor:** conceptualisation, resources, and project administration. **Matthew Flowers:** conceptualisation and resources. **Richard Lakeman:** resources and writing – review and editing. **Marie Hutchinson:** conceptualisation, methodology, resources, writing – review and editing, and supervision.

## Conflicts of Interest

The research was conducted, analysed, reported, interpreted and written independently of the organisations that awarded Esario IV Daguman's PhD scholarship funding. The views presented in this study are solely those of the authors and do not necessarily reflect those of the funding organisations.

## Supporting information


Data S1.

## Data Availability

The datasets are not publicly available due to the requirements outlined in the quality assurance and ethical approvals obtained.

## Endnote

^1^
The authors used ‘service users’ to refer to people who utilise services, including individuals who may not have the rights colloquially linked to a ‘patient’ or ‘consumer.’ The authors acknowledge the broader debate regarding language choice in mental health research.
