# Supplementary material for: Differentiating Therapeutic Responses That Reduce Restrictive Practice Use and Situational Aggression in an Acute Mental Health Unit
Source: J Clin Nurs. 2025 Mar 14;34(11):4698–709. doi: 10.1111/jocn.17727 (PMC12489444; doi:10.1111/jocn.17727)

**Supplementary Materials**

**Nursing Interventions Descriptions**

The acute mental health unit staff supported service users through the combination, or stand-alone use, of the following interventions. These intervention descriptions were based on nurses’ responses recorded in the logs.

Verbal de-escalation relates to using words and relational skills, including active listening, rapport-building, limit-setting, and problem-solving, to support a person in making sense of their mental health crisis, being brought to a safe space, and working towards their immediate needs and goals. In this intervention, the engagement is brief rather than long-term support.

Distraction is a technique that interrupts a situation and attracts the attention of a service user to something else, away from the source of distress. This intervention can involve shifting the focus of a conversation to external topics or other stimuli unrelated to the service user’s immediate concerns.

Sensory modulation refers to supporting access to uplifting, sensory-related experiences, including but not limited to music, musical instruments, weighted stuffed toys, and massage chairs. A key element is gustatory distraction, where food and drinks are used to shift focus through taste, such as offering a warm beverage or snack. It also covers supporting access to creative expression of oneself, such as colouring and art therapy.

Reality-basing or -testing means that a service user is supported to challenge the truth of their experiences, including guiding the person back to what is, and has been, happening. Wherever possible, the staff supports the person in understanding the relationship between themselves and their false beliefs, which clinicians would frame as delusions.

Change of environment involves bringing the person a sense of escape and release in a physical space that lessens worry about, and hyper-vigilance for, a mental health crisis. This intervention is exemplified by helping the person move to another area within the acute mental health unit, away from the physical source of distress.

Individualised staff time is an extended form of verbal de-escalation. It focuses on becoming physically available during a crisis, and includes supporting access to other non-relational and advanced relational interventions (or talk therapies). It supports a service user to develop self-management skills, including making a bed, attending to their hygiene, and enabling them to voice their frustrations.

Culturally-sensitive Care supports access to cultural experiences or encourages a service user to engage in spiritual activities. It also means that a person is supported to experience a sense of safety outside the clinical context.

Phone calls are telecommunication-assisted conversations of a service user with any family members, their legal aid, and individuals from services outside the acute mental health unit, such as calls to financial and banking services.

Hospital ground leave grants a service user permission to spend time outside the acute mental health unit, either escorted or on their own. The Mental Health Act 2007 (NSW) governs the basis for granting and reviewing leave requests.

## Silhouette plot for the number of optimal clusters


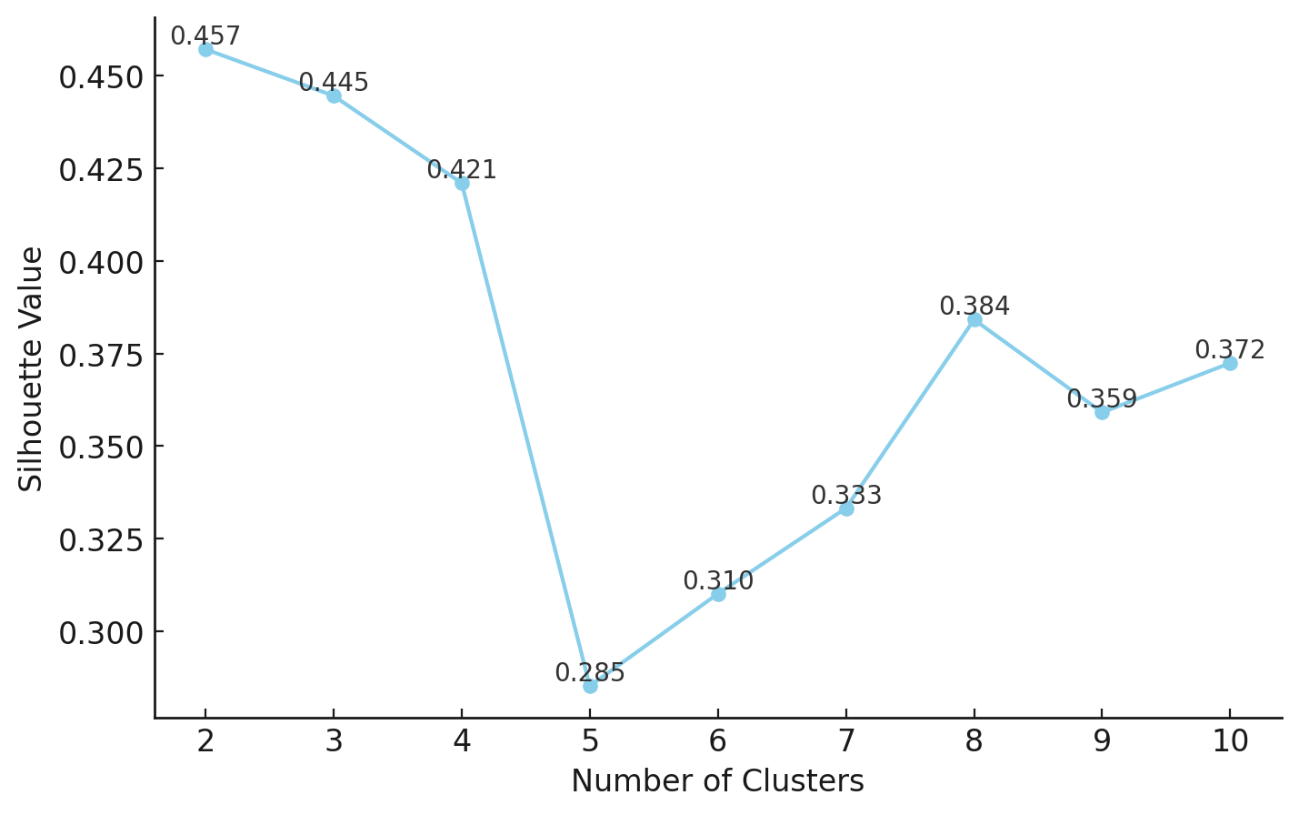

Supplement: Supplementary file 1 — Data S1. [file JOCN-34-4698-s001.docx]
